# LGBT in the Military: Policy Development in Sweden 1944–2014

**DOI:** 10.1007/s13178-015-0217-6

**Published:** 2016-02-04

**Authors:** Fia Sundevall, Alma Persson

**Affiliations:** Department of Economic History, Stockholm University, 106 91 Stockholm, Sweden; Department of Thematic Studies, Division of Gender Studies, Linköping University, 581 83 Linköping, Sweden

**Keywords:** LGBT, Policy, Armed forces, Discrimination, Working life, Military service, Sweden

## Abstract

This article contributes to the growing field of research on military LGBT policy development by exploring the case of Sweden, a non-NATO-member nation regarded as one of the most progressive in terms of the inclusion of LGBT personnel. Drawing on extensive archival work, the article shows that the story of LGBT policy development in the Swedish Armed Forces from 1944 to 2014 is one of long periods of status quo and relative silence, interrupted by leaps of rapid change, occasionally followed by the re-appearance of discriminatory policy. The analysis brings out two periods of significant change, 1971–1979 and 2000–2009, here described as turns in LGBT policy. During the first turn, the military medical regulation protocol’s recommendation to exempt gay men from military service was the key issue. During these years, homosexuality was classified as mental illness, but in the military context it was largely framed in terms of security threats, both on a national level (due to the risk of blackmail) and for the individual homosexual (due to the homophobic military environment). In the second turn, the focus was increasingly shifted from the LGBT individual to the structures, targeting the military organization itself. Furthermore, the analysis shows that there was no ban against LGBT people serving in the Swedish Armed Forces, but that ways of understanding and regulating sexual orientation and gender identity have nonetheless shaped the military organization in fundamental ways, and continue to do so.

## Introduction

In recent decades, policy on lesbian, gay, bisexual, and transgender (LGBT) service members has undergone dramatic transformations in numerous countries. Bans have been lifted, and discriminatory regulations have been replaced with more inclusive policies. There is now a growing body of literature on military LGBT policy and practice in NATO member countries, particularly the U.S., and a few of the major non-NATO allies, particularly Israel (see e.g., Basham 2013; Belkin 2012; Belkin and Levitt 2001; Britton and Williams 1995; Hekma 1991; Herbert 1998; Herek et al. 1996; Kaplan and Ben-Ari 2000; Lehring 2003; Rimmerman 1996; Trivette 2010).

A case of LGBT policy development in the military that has not been the focus of scholarly attention is Sweden. Today this non-aligned Scandinavian nation’s Armed Forces is regarded as one of the world’s most progressive in terms of inclusion of LGBT personnel (Polchar et al. 2014). Its military command marches for LGBT rights in the capital’s Pride parade and actively seek to recruit LGBT personnel in order to increase diversity in the ranks. Sweden also stands out historically as one of the first countries in the world to conscript and employ openly gay men. However, in spite of various steps towards inclusive policies since the 1970s, it seems that almost no LGBT people served openly in Sweden before the late 1990s. Furthermore, there has been a widespread and informally accepted homophobic jargon that is still present (Fahlstedt 2000, 2009; Forsberg et al. 2003; Persson 2012; Sundevall 2014).

This article represents the first scholarly effort to map out and analyze policy development on LGBT personnel in the Swedish Armed Forces (SAF). It shows how LGBT policy has developed in Sweden starting in 1944, when homosexual acts were de-criminalized, and analyzes how sexuality and gender identity has been described and regulated in military policy.

Previous research exploring sexuality in modern Swedish military history is sparse, primarily focusing either on the same-sex prostitution of soldiers in the late 1800s and early 1900s (Parikas 1999a, b; Sörensen 1998), or narratives and/or experiences of discrimination at the turn of the third millennium (Eriksson-Zetterquist et al. 2011; Fahlstedt 2000, 2009; Forsberg, et al. 2003; Sundevall 2014). The only literature to date on policy developments is a U.S. General Accounting Office report from the early 1990s (GAO 1993), which reviewed policy steps regarding homosexuals in the military in 25 nations, including Sweden.

We argue that the relevance of policy, practice, and discourse on LGBT in the military extends far beyond the military context itself. Ideals of gender and sexuality that are produced in the military are crucial for society at large, and should be situated in a broader historical and socio-cultural context of LGBT rights. By looking specifically at the military, we show how the social and legal status of LGBT people in Swedish working life and society has been re-negotiated, but also how tenacious patterns remain and re-appear. In so doing, we challenge a common narrative that portrays LGBT policy development in contemporary history as a linear progress, moving steadily from condemnation and exclusion to acceptance and inclusion.

The structure of the article is as follows: First, the theoretical framework is presented, followed by the methods of analysis, data collection and description of the source material. Next, an introduction to the Swedish case is provided, followed by an overview of LGBT policy developments in the SAF from 1944 to 2014. Subsequently, two phases of change are identified and analyzed. A concluding discussion then summarizes the main findings of the study.

## Theoretical and Methodological Framework

Our theoretical framework is based on a poststructuralist understanding of gender and sexuality, and the conviction that gender and sexuality are mutually constitutive categories that can never fully be disentangled from one another. Normative ideals of gender are intimately intertwined with norms about sexuality; more specifically, heterosexuality (e.g., Butler 1990; Connell 2009). In the military context, like many others, it becomes apparent that masculinity is intimately connected to, and co-constructed with, heteronormative ideals.

Gender relations are constructed in everyday social interaction, in tandem with the construction of norms around sexuality. Such a constructivist approach challenges the understanding of gender and sexuality as essential, homogenous, and stable categories. Rather, it analyzes how these categories are made sense of, i.e., how they are explained, negotiated, challenged, and defended in different social settings. In this social process, organizations are crucial as gendered, and gendering, institutions. Sociologists Elin Kvande and Bente Rasmussen (1993) describe organizations as the “melting pot or ‘transformer’ where society’s general perceptions and ideas of masculinity and femininity are produced” (p. 47, our translation from Swedish). We argue that this is the case also for the production of perceptions and ideas of sexuality.

In the study of gender and sexuality in social and labor history, the military provides a privileged empirical setting. Historically, the soldier has served as the “quintessential figure of masculinity” (Dawson 1994, p. 1). It was in the military that boys, according to the popular saying found in many languages, were made into men. Here, masculinity was (and is) actively and consciously constructed and consolidated, oftentimes in relation and in contrast to notions of femininity, as well as, in late modern history, homosexuality (Bulmer 2011; Connell 2009; Hearn and Parkin 1995; Herbert 1998; Higate 2003; Kronsell 2012).

During most of the period studied here, the SAF was the largest to second-largest state employer in the nation (SCB annual series). In addition, because of the conscription system, it was an organization where the great majority of Swedish men were forced to spend a year or more of their early adulthood, learning and performing military labor (cf Zürcher 2013). Restrictions against non-heterosexual service members both reflect and reproduce a heterosexual masculine ideal (Britton and Williams 1995). Thus, the military was, and is, closely connected to constructions, reconstructions and expressions of both masculinity and heteronormativity, and a site for complex interactions of gender, sexuality and power. Participating in armed defence, in many countries, has also been considered a key to full citizenship. As such, the military constitutes a highly politicized arena for LGBT rights and has therefore often been considered an important target for LGBT advocacy demanding equality. Scholarly debate theorizing on gender and sexuality in the military context, however, problematizes the ways in which LGBT rights, as well as women’s rights, are used to legitimize the military organization, states’ military spending, and western imperialism (e.g., Basham 2013; Puar 2007; Spade 2013).

Inspired by the work of Carol Lee Bacchi (1999, 2009), we use policy material to analyze the representation of LGBT in, and in relation to, the SAF. As Bacchi notes, “every policy proposal contains within it an explicit or implicit diagnosis of the ‘problem’” (1999, p. 1). There is a prescriptive element to policy material, in the sense that it provides a solution to a phenomenon, thereby defining it as a specific kind of problem (Bacchi 2012). In our historical contextualization of how sexuality and gender transgression has been described and regulated in the Swedish military, we show how representations of “the problems” of LGBT have been formulated, contested and re-articulated over time. We also show that there have been major shifts in representations during the studied time period, but that the process has not been one of stable development from exclusion to inclusion. Rather, we have found what Bacchi (2012), inspired by Foucault, calls “problematizing moments” (p. 2), i.e., specific times and places at which the understanding of sexuality and gender transgression was re-formulated, which we analyze as policy turns.

Throughout the article we use the contemporary term *LGBT*, while acknowledging that this calls for some consideration. Applying the term LGBT to identities and policies in the past can be considered problematic and ahistorical since it was coined in the latter part of the period in focus. Furthermore, there is a risk that the acronym conceals that debates, policy, and activist work on LGBT rights have primarily attended to the rights of lesbians and, in particular, gays, while bisexuals and transgender people have been marginalized and largely rendered invisible. While we have chosen to adhere to contemporary parlance we strive to make clear when the documents we explore do in fact deal with LGBT or with a particular group, identity, or conduct within this umbrella acronym.

### Source Material

At the onset of the project, very little had been written about the history of LGBT policy in the Swedish Armed Forces, and there was no compilation or analysis of key documents on the case. Therefore, outlining the developments demanded thorough investigations into a wide range of archives. Large numbers of printed and unprinted documents were thus explored in order to locate significant sources that could, in turn, point us in the direction of key policy documents and the context in which they were written. This included military medical manuals, government bills, parliamentary minutes, archival collections (from military divisions, subdivisions and organizations, as well as parliamentary commissions, public offices, and LGBT advocacy groups) and periodicals (including the official journals of the Armed Forces as well as of the Army, Marine and Air Force respectively, and the members’ journals of the Swedish Association of Military Officers and of the Swedish Federation for Sexual Equality). Unless otherwise stated, all quotes from the source material are translated from Swedish by the authors.

In addition to exploring when, how, and why policy changes occurred, and analyzing how matters of LGBT in the military were represented as problems, the source material was used as a means to pinpoint silences and status quo. When exploring the source material, we were not only looking for what was said, how or when, but also what was left out and/or rendered unproblematized.

## Military Structure and LGBT Public Policy in Sweden

Before turning to our findings, we will provide an introduction to the Swedish case by highlighting some significant aspects of, first, the structure of the Swedish Armed Forces, and, second, some key features of Swedish LGBT public policy developments since the 1940s, with a primary focus on developments concerning anti-discrimination legislation.

### The Swedish Armed Forces

Since the late 1800s, Sweden’s official security policy has emphasized the nation’s non-participation in military alliances during peacetime, aiming at neutrality in the event of war. Following the reorganization of the SAF at the turn of the millennium, from an invasion-based defense organization to an increasingly international and mission-based one, its transnational military cooperation has increased and now includes active cooperation with, e.g., NATO on international missions and participation in the EU battlegroup unit (Egnell et al. 2014).

The Supreme Commander, in NATO terminology *Chief of Defence*, is the authoritative head and central supervisor of the SAF, reporting to the Swedish government which is the highest executive authority of the SAF. The SAF of today is made up of three parts: contract units, standing units, and the Home Guard, all recruited on a volunteer basis. During most of the period covered in this study, however, a substantial part of the SAF personnel were enlisted through the system of conscription of male citizens. Women were thereby indirectly barred from all military—and most of the so-called civil-military—positions (paid as well as unpaid) within the SAF until the 1980s, when they were granted formal access to the same qualifying training as conscripted men, as well as the right to serve in all positions and branches of the SAF. In 2010, the Swedish parliament abolished general male conscription, removing the last remaining formal demarcation between women and men in its personnel recruitment system (Sundevall 2011).

### LGBT Public Policy Developments in Sweden Since 1944

During the period in focus in this article Sweden, like many other European nations at the time, adopted a number of public policies strengthening LGBT people’s rights. In terms of lesbian, gay and bisexuals’ rights, a key policy step was taken in 1944 when same-sex sexual relations—or *fornication against nature* as it had been referred to in Swedish law since 1864—were de-criminalized, following a redefinition in public debate of homosexuals as mentally ill rather than sinners (Lennerhed 2002; Rydström 2003). While the de-criminalization put an end to prosecution on the ground of same-sex sexual relations, homosexuality remained the subject of extensive social stigma, with homophobia growing in the 1950s (Lennerhed 1994; Rydström 2007, 2012).

Entering the 1970s, the nation’s largest and most influential gay and lesbian rights activist organization, the RFSL (the Swedish Federation for Sexual Equality)—founded in 1950—was politicized and intensified its struggle against discrimination. As a direct result of efforts by the RFSL, a large majority of the members of parliament agreed in 1973 on a principal statement declaring that “from society’s point of view, a relationship between two persons of the same sex is a fully acceptable form of living together” (LU 1973:30, translation by Rydström 2007, p. 206). In the fall of 1979, 25 years after the de-criminalization, homosexuality was de-medicalized when it was removed from the Swedish Classification of Diseases (Socialstyrelsen 1979). Meanwhile, a commission had been appointed by the government to “propose measures which are needed in order to remove any remaining discrimination of homosexuals” as well as “compile and give an account of available scientific documentation about homosexuality” (SOU 1984:63, p 29, translation by Rydström 2007, p. 207). The proposals of the commission would a few years later result in the addition of “homosexual orientation” to the grounds of discrimination prohibited in the Swedish penal code (SFS 1987:610), as well as a new act on unmarried cohabitees, giving cohabitees in same-sex and opposite-sex partnership the same legal status (Rydström 2004). In the late 1990s, parliament further strengthened and supplemented previous anti-discrimination acts. In tandem, a new public office—the Ombudsman against Discrimination on the Basis of Sexual Orientation (officially abbreviated “HomO”, pun intended)–was established in order to monitor and promote compliance with the newly approved Anti-Discrimination Act (Rydström 2000).

During this period, transgender rights were to a large extent marginalized. While Sweden became the first nation in the world to allow the legal change of gender identity in 1972, it took another three decades before transgender rights began to climb the parliamentary agenda and the RFSL engaged in the struggle (Rydström 2004). In 2008, “transgender identity and expressions” was added to the new Anti-Discrimination Act, which took effect the following year, and in 2013 the requirement for sterilization in order for a person to change their legal gender was abolished after years of activist work (Parliament 2013).

## LGBT and the Swedish Military: An Overview

Beginning with the de-criminalization of same-sex sexual relations in 1944 and concluding with the Supreme Commander and the Minister of Defense marching together for LGBT rights in the Pride parade in the nation’s capital 70 years later, Table 1 summarizes internal and external policy developments relating to the exclusion and inclusion of LGBT people in the SAF. It includes policy steps of formal and legislative, as well as symbolic, character.

**Table 1:**
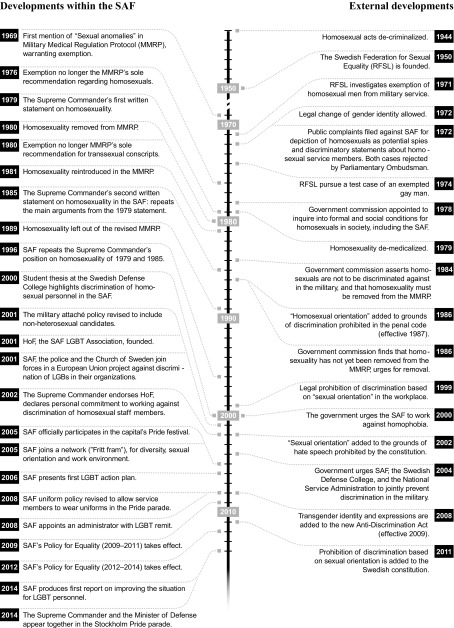
LGBT and the Swedish military: an overview of policy developments 1944–2014

As Table 1 shows, policy on LGBT SAF personnel underwent radical transformations in the late twentieth and early twenty-first centuries. However, as the table highlights, this was not a process of liberal linear progression, moving steadily from exclusion and discrimination to inclusion and diversity. Rather, we argue that key policy changes took place during two quite distinct time periods, 1971–1979 and 2001–2009, described in the following as policy turns. These policy turns are analytical constructs, characterized by an increased intensity in policymaking activity. During both of them, the established ways of representing homosexuality as a problem were dislocated and re-negotiated in significant ways.

## The First Turn in SAF LGBT Policy (1971–1979)

A significant finding is that in Sweden, unlike many other nations (see e.g., Gade et al. 1996), there was no ban on LGBT personnel in its Armed Forces. There was, however, a military guideline that partly worked to that effect, as well as formal and informal policies excluding LGBT service members from certain positions. The main issue at stake during the first turn in SAF policy on LGBT personnel was the assessment criteria for suitability to serve in the military. With very few exceptions, the debate during this period concerned gay men, while lesbians and transgender people (be they lesbian, gay, bisexual or heterosexual) were largely left out of the policymaking agenda.

The greatest challenges during this period were posed by the RFSL. Beginning in 1971, the RFSL called into question a number of SAF matters, including an alleged statement by the Supreme Commander disapproving of homosexuals in commanding positions (RFSL 1972a) and the depiction of homosexuals as potential spies in a booklet produced by the police, Security Services and the Armed Forces in a joint action (*Säkerhetsupplysning*^1971^; RFSL 1972b). Its main target, however, was the normative and discriminatory guidelines of the Military Medical Regulation Protocol (MMRP), the only policy document at the time regulating LGBT in the SAF.

Until the late 1980s, the MMRP–produced by the Defense Medical Administration Services (Försvarets sjukvårdsstyrelse, FSS)–was the core document for the assessment of conscripts’ physical and mental health and their suitability for military service. From 1969, when a new and considerably more detailed version was introduced, until 1976, the MMRP advised that conscripts diagnosed with homosexuality, transsexuality or other “sexual anomalies” (1978, p. 225) should be exempted from service or considered for service solely in a non-military (civilian) subdivision of the defense organization. In practice, the guidelines were not always strictly enforced (RFSL 1972c; SOU 1984:63), and there are reports from both within and outside of the SAF of homosexual and transgendered service members serving more or less openly (see e.g., *Hälso- och sjukvård vid Försvaret*^1970^–1975; Kottenhoff 1971; Nygren 1969). However, there are strong indications that these cases were exceptions and that the most common practice was to exempt conscripts diagnosed with sexual anomalies (RFSL 1972c; VKS 1974). In addition to serving as a guide in the process of assessing conscripts, we argue that the MMRP was important in a wider symbolic sense, reproducing and legitimizing the SAF’s institutionalized heteronormativity.

As a result of RFSL’s work, including successfully pursuing a case of principle against the National Service Administration in 1974 when a gay man was exempted from military service against his will (RFSL 1974; FSS 1974), the MMRP’s recommendations on so called sexual anomalies were revised, first in 1976 and then again in 1980. The 1976 revision entailed that homosexuals as well as transsexuals could be considered for service in various military divisions, although transsexualism was considered a somewhat greater obstacle than homosexuality. The 1980 revision, in turn, removed homosexuality from the MMRP with reference to its removal from the Swedish Classification of Diseases in the previous fall (FSS 1980). Meanwhile, the new MMRP (1980) recommendation on transsexuality gave the examining physician the option to rule that the transsexuality was non-significant for the conscript’s ability to serve.

In 1979, the Supreme Commander issued the first official statement on homosexuality in the SAF (ÖB 1979). In the statement, he described a general view on homosexuals in society at large, and the particular working environment of the military. The responsibility of managing both of these aspects was assigned to the homosexual individual:

What is crucial is the individual’s capacity to deal with (that is, accept, control and find an outlet for) his sexuality. For a mature individual for whom, for example, homosexuality or other so-called deviant behavior is an accepted and controlled part of his personality, this behavior is no grounds for special treatment in the Armed Forces. (p. 1)

Accepting rather than hiding one’s sexuality was considered particularly important for officers in senior positions, since an officer wishing to conceal his homosexuality was considered a potential security threat due to risks of blackmail. In line with the general debate, this statement only applied to homosexual men. At this time, bisexuality was never discussed, and neither were issues concerning gender identity. Lesbians were not addressed at all, since women were not yet allowed to serve as conscripts or officers.

The Supreme Commander’s first policy statement marks the end of the first turn in SAF LGBT policy, a period in which homosexuality in the military had been challenged and re-negotiated in important ways. The process of abolishing the discriminatory practices in the SAF represented by the MMRP came with an epilogue, including a twist. Referring to the need for documentation, the Defense Medical Administration Services reintroduced homosexuality into the MMRP in 1981, effective immediately (FSS 1981). Following complaints from the RFSL, the FSS then announced that it would once again remove the diagnosis when it issued the next MMRP (FSS 1983). However, that edition was delayed until 1989 (FMK 1989. See also SOU 1984:63, 1986:43). This means that, formally speaking, homosexuality remained a diagnosis within the SAF, and could be used to exclude gay men from military education, training and work, for another 10 years after the classification was removed from the Swedish Classification of Diseases.

During this first turn in LGBT policy development in the SAF, the arguments used to legitimize the exclusion of homosexual men, enabled primarily through the MMRP, revolved around notions of security and threat in different ways. Three partly overlapping ways of representing homosexuality as a problem emerge from the analysis.

The first was based on the idea of the gay man as a potential threat to national security. It coincided with the perceived threat from foreign nations and the fear that they would gain access to classified military information. As in many other nations at the time (e.g., Lewis 2001), gay military personnel in general, and gay commanding officers in particular, were perceived as high-risk targets of blackmail attempts (e.g., *Säkerhetsupplysning*^1971^). Based on such arguments, an openly gay soldier or officer would not have been considered a threat to national security since the risk was connected to a person’s wish to conceal his homosexuality. Such considerations were, however, not addressed.

The second way of representing homosexuality as a problem was framed as a matter of security; not for the nation but rather for the gay man himself. It was expressed as a concern for the individual gay soldier’s mental and physical health, related to anticipated exposure to harassment from other conscripts, and/or difficulties adjusting to the group. While disclosing one’s non-heterosexuality was implied to reduce the risk of blackmail, it was nevertheless considered to make the individual a potential target of harassment. It could, therefore, in the words of the Defense Medical Administration Services (FSS 1974), be “inhumane” (p. 2) to draft homosexuals. The particular and “extremely masculine” (VKV 1971) military work environment was pinpointed as an essential part of the problem, creating personal difficulties and “unnecessary tragedies” (Ibid) for the gay soldier.

The third way of representing homosexuality as a problem was less explicit than the former two. It circled around a perceived need to protect other, assumed to be heterosexual, conscripts—particularly the gay conscript’s potential subordinates and/or “less independent peers” (FSS 1974, p. 2)—from homosexual colleagues. These kinds of arguments drew upon a stereotypization of gay men as sexual predators, and the prevailing notion that homosexuality might spread through seduction. As noted by Rydström (2007), the seduction theory was highly influential in Swedish politics on homosexuality in the mid-1900s, and continued to influence legislation until the end of the 1970s.

Although the SAF identified various aspects of the military environment and culture as the cause of problems for gay conscripts, it primarily placed the responsibility for dealing with these problems outside the military and rather on society and the individual gay conscript. Hence, the alternative of the SAF working to create a more inclusive work environment was largely left unattended to. Nevertheless, some steps towards changing attitudes within the ranks were taken by the SAF in 1979. These initiatives were focused on “creating understanding of and tolerance for (different) forms of deviancies” (ÖB 1979, p. 2), hence reconstructing homosexuals as the (sexual) other while leaving the heteronormative foundations of the SAF unchallenged.

## The Second Turn in SAF LGBT Policy (2001–2009)

The first turn ended with the Supreme Commander’s first written statement on homosexuality in 1979, and the removal of homosexuality from the MMRP. From that point, not much happened in terms of SAF LGBT policy for more than two decades. During the 1980s and 1990s, new written statements were issued, repeating the content of the 1979 policy (ÖB 1985; SAF 1996). During these years, very few LGBT people seem to have served openly in the SAF (although some were indeed serving), and there was a relative silence on questions regarding sexual orientation as well as gender identity.

After a long period of status quo, a new phase of SAF LGBT policy development began as the SAF responded to external and internal pressure to address formal and informal discrimination within the ranks. Like the first turn in SAF LGBT policy, the second one was closely connected to the development of LGBT rights in society at large. Once things started moving, the change was rapid. Entering the second turn, formal inclusion once again became a matter of negotiation when a discriminatory SAF policy on the recruitment and selection of military attachés, which limited the position to married heterosexual men, was brought to the attention of the Parliamentary Ombudsman in 2001 (HomO 2001). Following a joint action of the Parliamentary Ombudsman offices on Gender Equality (JämO) and against Discrimination on the Basis of Sexual Orientation (HomO), the SAF altered the policy (Ibid), hence doing away with the last remnant of formal discriminatory policy against lesbian, gay, and bisexual personnel in the SAF. From this point on, formal discriminatory policy was no longer at stake and the focus shifted to other aspects of discrimination and new representations of homosexuality as a problem.

In parallel with the military attaché case, the situation for homosexuals in the Armed Forces was also addressed from various other parties. In 2001, the (Social-Democratic) government urged the SAF to actively work against harassment caused by homophobia (Fö 2000), and to “put the issue of homosexuals on the agenda and discuss it more openly” (Gov. 2001, p. 60). Around the same time, the first academic study on the discrimination of homosexuals within the SAF (Fahlstedt 2000) caught the attention of journalists and members of parliament (e.g., Berg 2000; Hermansson 2001; Motion 2001/02:Fö266; Poohl and Ekman 2001; *Sydsvenskan*^2000^).

The study, conducted by Captain Krister Fahlstedt (2000) while enrolled in the National Defense College’s officer’s program, highlighted homosexual personnel’s experiences of discrimination, and stressed the need for the SAF to address the problems. In the fall of 2001, Fahlstedt, himself an openly gay man, turned to the op-ed pages of the SAF’s personnel magazine, calling for the SAF to break the silence on homosexuals in the forces, and for LGBT personnel to come together in an association (Fahlstedt 2001). Before the end of the year an LGBT Association had been formed within the SAF, and shortly thereafter the Supreme Commander called for a press conference to express his support for the association and declare the need for the SAF to work more actively to combat harassment and prejudice within the ranks (SAF 2002).

The problem of discriminatory practices against homosexuals in the SAF was framed by Supreme Commander Hederstedt in a way similar to how the Supreme Commander described it in 1979: as a reflection of social prejudice against the group, and hence not an SAF problem per se. But while his predecessor had primarily placed responsibility for managing the problems on society as well as the individual homosexual, Hederstedt underlined the need for a structural approach rather than an individual one. “We must take our responsibility as an employer and bring about a change of attitude”, he declared, and committed to getting personally involved should any case of sexual orientation discrimination or harassment within the SAF come to his attention (SAF 2002, p. 1).

All these transformations—the military attaché policy, the government’s instructions to actively work against homophobia, Fahlstedt’s essay, the formation of the LGBT Association and the Supreme Commander’s public declaration of his support for LGBT rights in the SAF—all took place within the course of 1 year. After this, a number of measures were taken by the SAF in close cooperation with its LGBT Association to investigate and tackle homophobia, increase visibility, and promote diversity within the ranks. This included, but was not limited to, a joint action with the Church of Sweden and the national police force—organizations which also had a reputation of being a hostile working environment for anyone diverging from a certain male heterosexual standard—to address and combat homophobia in their workplaces by means of education, information, and research (Bildt 2004; Fahlstedt 2009; Forsberg et al. 2003; Normgiving diversity 2004). From 2005 on, the SAF also took active part in the capital’s yearly Pride festival (Sjödén 2005), and in 2008 it appointed an LGBT advisor—the first ever in a military organization, according to the SAF itself (2008a)—to coordinate and monitor the SAF LGBT inclusion initiatives.

Whereas words were followed up by actions regarding lesbian, gay and bisexual personnel, transgender personnel were still largely rendered invisible. This slowly started changing towards the end of the second turn, mirroring then-current policy debates in society at large and the parliament’s passing of a new Discrimination Act (SFS 2008:567), which for the first time prohibited discrimination based on transgender identity or expression.

The SAF’s new self-perception as an agent of change was not limited to the organization itself. As one of the largest state employers, also training large numbers of conscripts every year, the SAF considered itself to have “an important normative role in society” (SAF 2009 p. 4). The revision of the uniform policy, which enabled personnel to wear their uniforms when attending Pride parades, included a similar argument and extended this responsibility beyond the Swedish context. Through the increased focus on international peacekeeping missions, notions of justice and democracy in a wider sense were included. In a 2008 SAF press release on a revision of uniform policy, it was stated that this would help Sweden and its Armed Forces to “take the lead internationally [….] and act as a forerunner nation in LGBT matters” (2008b, p. 1). This rationale should be understood in relation to the Swedish national self-image as an international forerunner in human rights’ advocacy (see e.g., Ministry of Foreign Affairs 2013). As the defender of the nation and crucial actor in Sweden’s international peacekeeping operations, the Armed Forces thus assumed a key role in the symbolic demonstration of this self-image.

Following the enactment of the Discrimination Act, the SAF revised its policy document on equality (2009) to address “transgender identity or expression” and “sexual orientation” along with the other five grounds of discrimination (gender, ethnicity, disability, religious or other belief, and age) included in the new act. The policy targeted structural and organizational obstacles to equal opportunities, and declared diversity to be “a source of strength” (p. 3) for the SAF, contributing to the organization’s efficiency. In the section on LGBT, it was further stated as a goal of the SAF to have “a working climate where no-one has to conceal their sexual orientation or gender identity against their will”, and that it was therefore of great importance, to make active efforts in order to “counteract every instance of discrimination and harassment” (p. 6). This mainstreaming of LGBT rights into the general equality policy of the SAF marks the end of the second turn in SAF LGBT policy development.

Summarizing the second turn, the dominant representation of homosexuality as a problem was no longer the perceived risk of including “sexual deviants” in the military. Rather, what was now targeted was the structural and organizational problem of homophobia and heteronormativity in the ranks. The solution was described as increased LGBT competence among staff (through education initiatives) and a public display of tolerance and openness (through, e.g., participation in the capital’s Pride festival). Hence, the problem was no longer represented as the LGBT individual subject or prejudice in society, but rather the military organization itself, and the SAF increasingly assumed a pro-active role in fighting discrimination and harassment against its LGB and, with time also T, personnel.

## Concluding Remarks

This article has outlined LGBT policy developments in the Swedish military, from 1944 and the de-criminalization of homosexual acts, to 2014 when the Supreme Commander and the Minister of Defense marched together in the Stockholm Pride parade. During these seven decades there was an overall transformation from the partial exclusion of LGBT personnel, regulated by the MMRP and other more or less formalised policies, towards a more inclusive organization with a formal policy that condemns discrimination based on sexuality or gender identity. This process was characterized by periods of rapid transformation during which the representations of problems regarding homosexual service members were more intensely re-negotiated, and important policy development followed. These periods are described here as turns in LGBT policy development, and we argue that the two crucial turns during the period studied took place in 1971–1979 and in 2001–2009.

During the first turn, the MMRP and its recommendation to exempt non-heterosexual men from service formed the main target of contestation. The turn ended with the Supreme Commander’s first ever statement on homosexual service members, and the removal of homosexuality from the MMRP. During the second turn, there were remnants of formal discriminatory policy that were called into question and abolished, but the main area of contestation was the invisibility of LGBT staff members and the heteronormative and homophobic working and training environment of the SAF. The representations of homosexuality as a problem were fundamentally different in each of these turns. The first was based on homosexuality as risk: to national security, to the individual gay soldier, and to his potentially susceptible peers and subordinates. The prejudice of society at large added to the representation of homosexuals as a problem in the military. In the second turn, the military itself was the target of change. The representation of the problem was no longer the LGBT service members, but rather the heteronormative environment and the lack of what the SAF referred to as LGBT competence. At this point, prejudice in society domestically and internationally was described as something the SAF could and must combat.

While the main narrative moves from extensive discrimination supported by Armed Forces policy, to an organization that participates in the Pride parade and actively seeks to recruit LGBT staff in order to increase its diversity, there is a need for a more nuanced account. During the period in focus here, the SAF has not been in a steady state of progression towards LGBT policy inclusiveness. The back-and-forth process of removing and restoring homosexuality as a diagnosis in the MMRP during the 1970s and early 1980s illustrates the ambiguous and non-linear process of LGBT policy development in the SAF. The periods of few or no policy activities also need to be addressed. It is particularly noteworthy that the 1980s and 1990s saw little attention to matters of LGBT personnel in the SAF, and almost no policy development on the matter. To some extent, this mirrors the relative stagnation in Swedish LGBT policy development on an aggregated level during part of the time period in question. Hence, the story of LGBT policy development in the SAF is one of long periods of status quo, interrupted by leaps of rapid change, occasionally followed by the re-appearance of discriminatory policy.

Moreover, questions of policy versus practice must be taken into account. Although explicitly condemned by the Joint Chiefs of Staff, exclusionary practices such as homophobic jargon remain to this day. Inclusive policy, hence, by no means guarantees an inclusive approach in everyday SAF working life and training. Formal policy has been the focus in this article, and although there are irregularities in the linear progression, the main result is that the SAF’s LGBT policy has been thoroughly transformed over the seven decades in focus. Discriminatory policy has been replaced by equal-opportunity policy and the vision of a diverse military. Had we studied the implementation of policy, and the experiences of LGBT staff in the SAF, our results would likely have been much more ambiguous. Research indicates continued problems with a work environment where homophobia and heteronormativity abound. Indeed, homophobia and transphobia were common features of military life well beyond the second turn covered here (Forsberg et al. 2003; Persson, 2011; Sundevall 2014), making LGBT staff members camouflage their gender identity and/or sexuality for fear of negative or hostile reactions from peers and superiors (Bildt 2004; Fahlstedt 2009; Forsberg, et al. 2003).

An additional challenge is posed through the transition towards an increasingly international military. Such a transformation comes with new challenges for all SAF staff, but even more so for LGBT employees. They risk not only being sent on missions in countries where same-sex sexual relations and/or transsexuality are considered a crime, but also working side by side with soldiers from nations where it is difficult or impossible to serve openly (Fahlstedt 2009). This means that openness must be reconsidered and in some cases restricted. LGBT staff has called on the SAF to acknowledge these difficulties, and work more actively to support its LGBT soldiers and officers in their international missions. It is only recently (SAF 2014) that such challenges have begun to be addressed.
